# Towards delineating the chain of events that cause premature senescence in the accelerated aging syndrome Hutchinson–Gilford progeria (HGPS)

**DOI:** 10.1042/BST20190882

**Published:** 2020-06-15

**Authors:** Oliver Dreesen

**Affiliations:** Cell Ageing Laboratory, Skin Research Institute of Singapore, 8A Biomedical Grove, #06-06 Immunos, 138648 Singapore

**Keywords:** lamin A, progeria, senescence

## Abstract

The metazoan nucleus is equipped with a meshwork of intermediate filament proteins called the A- and B-type lamins. Lamins lie beneath the inner nuclear membrane and serve as a nexus to maintain the architectural integrity of the nucleus, chromatin organization, DNA repair and replication and to regulate nucleocytoplasmic transport. Perturbations or mutations in various components of the nuclear lamina result in a large spectrum of human diseases collectively called laminopathies. One of the most well-characterized laminopathies is Hutchinson–Gilford progeria (HGPS), a rare segmental premature aging syndrome that resembles many features of normal human aging. HGPS patients exhibit alopecia, skin abnormalities, osteoporosis and succumb to cardiovascular complications in their teens. HGPS is caused by a mutation in *LMNA*, resulting in a mutated form of lamin A, termed progerin. Progerin expression results in a myriad of cellular phenotypes including abnormal nuclear morphology, loss of peripheral heterochromatin, transcriptional changes, DNA replication defects, DNA damage and premature cellular senescence. A key challenge is to elucidate how these different phenotypes are causally and mechanistically linked. In this mini-review, we highlight some key findings and present a model on how progerin-induced phenotypes may be temporally and mechanistically linked.

## Introduction

Aging can be defined as a gradual deterioration of cell and tissue function, resulting in an elevated risk of developing a large number of chronic illnesses. These include cardiovascular disease, diabetes, decreased cognitive and hearing ability, neurodegenerative diseases, and loss of bone and muscle mass. Aging is also correlated with an elevated cancer risk. On a cellular level, increased levels of DNA damage, genomic instability, epigenetic alterations including loss of heterochromatin, telomere shortening, mitochondrial dysfunction, impaired nutrient sensing, loss of proteostasis and permanent growth arrest (senescence) are all aging-associated phenomena [1]. However, given the complexity of the aging phenotype, and the ethical and practical limitations of longitudinal studies, little is known about how all of these ‘hallmarks of aging' are causally connected. Progeroid syndromes, which are rare premature aging disorders, may, however, offer a unique window on to the mechanisms that contribute to human physiological aging [2–5]. Progeria patients exhibit characteristic features of normal aging. Typically, these are variously represented by cardiovascular disease, osteoporosis, lipodystrophy, alopecia and aberrant skin pigmentation. Depending upon the type and severity of the particular syndrome, patients may succumb as early as in their mid-teens or may survive until their fifth decade. Progeroid syndromes can be classified into two main groups: The first group is caused by defects in DNA repair pathways or impaired telomere maintenance and includes Werner syndrome (WS), xeroderma pigmentosum (XP), Bloom syndrome (BS), Nijmegen breakage syndrome (NBS), Cockayne Syndrome (CS), Rothmund–Thomson syndrome (RTS), ataxia telangiectasia (AT), Fanconi anemia (FA), dyskeratosis congenita (DC) and Hoyeraal–Hreidarsson syndrome (HHS). The second group is caused by mutations in components of the nuclear lamina and is represented by Hutchinson–Gilford progeria syndrome (HGPS), restrictive dermopathy (RD), Néstor–Guillermo progeria syndrome (NGPS) and mandibuloacral dysplasia (MAD) [6–8]. In this mini-review, we will focus mainly on HGPS as it is arguably the best-studied progeroid syndrome.

## The nuclear lamina

A defining feature of all eukaryotic cells is the nucleus, a compartment encapsulated by a pair of membranes that are separated by the perinuclear space. The outer nuclear membrane (ONM) faces the cytoplasm, is contiguous with the endoplasmic reticulum and serves as an anchor point for the cytoskeleton, whilst the inner nuclear membrane (INM) faces the nuclear lamina; a ∼15 nm thick proteinaceous meshwork consisting of type V intermediate filament proteins [9–12] known as A- and B-type lamins. The ONM and INM are connected where they are spanned by nuclear pore complexes, massive channels that facilitate the nuclear-cytoplasmic transport of macromolecular cargoes. The INM and the lamina are connected through interactions with multiple integral membrane proteins. Prominent among these are a family of INM proteins, that share a 40 amino acid *L*AP2-*e*merin-*M*AN1 (LEM) domain, that binds both the nuclear lamina and repressed chromatin [13]. Most LEM-family proteins, including LAP2β, LEMD1 and Emerin are tethered to the INM via a single C-terminal transmembrane domain; they interact with each other as well as with the A- and B-type lamin proteins of the nuclear lamina.

The basic structure of the nuclear lamins features a ∼30 amino acid non-helical amino terminal head domain and a central α-helical coiled-coil rod domain of ∼45 nm. The rod domain is followed by a highly conserved immunoglobulin-like fold (Ig fold). Sequences downstream of the Ig fold domain are largely unstructured and, with the exception of lamins C and C2, terminate in a CaaX motif (C, cysteine; a, aliphatic amino acid; X, any amino acid but usually methionine) [9,11,14,15], the function of which is described in the next section. In contrast with their cytoplasmic intermediate filament counterparts, all members of the lamin family possess a classical SV-40-type nuclear localization sequence that is situated between the central alpha-helical rod and Ig fold domains.

As members of the intermediate filament family, both the A- and B-type lamins will assemble to form tetrameric filaments. These in turn may associate to form the higher-order nuclear lamina structure that is essential for the maintenance of nuclear architecture and stability. The A-type lamins are encoded by a single gene, *LMNA*, and include the major isoforms lamin A and lamin C, as well as minor isoforms such as CΔ10 and C2 [16]. The B-type lamins, lamin B1 and lamin B2, are encoded by two separate genes, *LMNB1* and *LMNB2*, respectively. The latter also encodes the germ cell-specific lamin B3 as a splice variant of lamin B2. The fact that lamins are notoriously insoluble, form filaments and lack any catalytic activity led to the initial notion that lamins function primarily to maintain nuclear shape and integrity. However, research in the past 20 years demonstrated that lamins play a crucial role in various cellular processes including mechanotransduction, modulation of nuclear stiffness and elasticity, chromatin organization, DNA replication and repair, and transcriptional regulation [4,17,18].

The composition of the lamina changes during embryonic development and, even in adults, there are obvious differences according to tissue type. Pioneering studies 30 years ago revealed that lamins are differentially expressed. B-type lamins are expressed in embryonic stem cells, throughout embryonic development and in all somatic cell types. In contrast, expression of A-type lamins commences midway through gestation and may vary significantly between different somatic cell types [19–22]. However, we still lack a complete picture of the precise composition of the nuclear lamina, as well as the stoichiometry of its components, in different human tissues. This is important as the differential expression of lamins—or lamina-associated factors—may explain how mutations or perturbations of the lamina give rise to such a large spectrum of different diseases, and why certain tissues are not affected [21–23].

## Processing of lamins

Soon after translation, and prior to assembly into the lamina, the C-terminus of lamins A, B1 and B2 undergoes many sequential modification steps. The first of these is the addition of a farnesyl group onto the cysteine residue of the C-terminal CaaX motif by a farnesyltransferase (FTase), the linkage consisting of a thioether bond. Farnesylation is followed by cleavage of the terminal-aaX tripeptide domain by the Zinc metallopeptidase *FACE1*/ZMPSTE24 or *FACE2*/Rce1. The newly exposed farnesylcysteine is then carboxymethylated by the isoprenyl-cysteine-carboxy-methyltransferase (ICMT) [24,25]. The hydrophobic farnesyl tail facilitates the interaction of newly synthesized lamins to the INM [26]. Finally, in the case of lamin A only, the terminal 15 amino acids, including the farnesylcysteine α-methyl ester are cleaved again by ZMPSTE24 resulting in the generation of mature non-farnesylated lamin A. This non-membrane bound mature lamin A has both lamina-associated and nucleoplasmic populations [27]. Neither lamin B1 nor B2 undergo this final processing step and instead both remain permanently farnesylated and tethered to the INM. Blocking lamin A farnesylation by mutating its C-terminal cysteine residue to a serine, and removal of its nuclear localization signal result in cytosolic accumulation of lamin A [28]. Inadequate processing of lamin A results in many devastating human diseases including HGPS, RD and MAD [29].

## Hutchinson–Gilford progeria syndrome

HGPS is a rare, autosomal dominant premature aging syndrome that affects roughly 1 in 4–8 million newborns. Patients are born without any overt clinical features, but develop signs of aging 18–24 months after birth. These include failure to thrive, alopecia, thin and wrinkled skin, aberrant pigmentation, loss of subcutaneous fat, stiffening of joints and weakened bone structure [8]. HGPS is fatal and patients generally die in their mid-teens as a result of cardiovascular complications [2,30]. Clinical examination of individuals with HGPS revealed a loss of vascular smooth muscle cells, aortic calcification, accumulation of atherosclerotic plaques and adventitial thickening. Collectively, these cardiovascular alterations result in myocardial infarction and stroke and are the main causes of death in progeria patients [2,8,30]. Two independent groups discovered in 2003 that HGPS is caused by a *de novo* heterozygous point mutation (1824C > T) in the *LMNA* gene [31,32]. This point mutation activates a cryptic splice donor site that results in the deletion of a 50 amino acid segment, including the second ZMPSTE24 cleavage recognition site, and results in the formation of a truncated and permanently farnesylated and carboxymethylated mutant form of lamin A, termed progerin.

## Atypical progeroid syndromes: restrictive dermopathy and mandibuloacral dysplasia

RD and MAD are caused by mutations in *ZMPSTE24* that result in partial or complete loss of enzymatic function, or mutations in the ZMPSTE24 proteolytic cleavage site in lamin A, thereby resulting in the accumulation of aberrantly processed and permanently farnesylated lamin A [33]. RD is characterized by clinical features before birth, including severe intrauterine growth defects, reduced fetal movement and perinatal lethality. The skin of RD patients is thin, translucent, tightly adherent, lacks elastic fibers and exhibits disorganized collagen organization. MAD can be classified into type A and B: mutations in lamin A that affect processing by ZMPSTE24 belong to type A, whilst mutations that impair the proteolytic activity of ZMPSTE24 are classified as type B. Both types share clinical features that resemble progeria but vary in severity; whilst patients with progeria display signs of aging 1–2 years after birth, patients with MAD generally develop abnormalities around the age of ∼4–5 years. These include skeletal abnormalities, partial alopecia, insulin resistance, lipodystrophy and abnormal skin pigmentation [34]. In conclusion, RD, HGPS and MAD are all caused by an accumulation of permanently farnesylated lamin A.

## Cellular phenotypes

HGPS patient-derived fibroblasts and various cell-based experimental systems have greatly contributed to our understanding how lamin A mutants perturb cell physiology and function. Original studies revealed that HGPS patient cells and various human cells expressing progerin develop nuclear abnormalities, loss of peripheral heterochromatin and a thickening of the nuclear lamina [35–37]. In addition, progeric cells display a persistent activation of the DNA damage response (DDR) checkpoints, telomere shortening, increased reactive oxygen species (ROS), genomic instability, impaired proliferation and premature cellular senescence [23,38–42]. What remains unknown is how these different phenotypes are mechanistically and/or causally linked, and whether they are a cause or a consequence of senescence.

### Nuclear abnormalities

The presence of the permanently farnesylated and INM-anchored progerin protein results in a thickening of the nuclear lamina, nuclear shape alterations such as invaginations and prominent lobulations, and clustering of nuclear pore complexes [35]. As a result of these nuclear envelop perturbations, HGPS nuclei are stiffer and more resistant to mechanical forces [43]. Contrasting these observations, nuclei from *Lmna*-deficient mice are softer and less resistant to mechanical stress [44]. Taken together, progeric nuclei may respond differently to physical stress than their normal counterparts. This may be particularly significant in those tissues that are subjected to constant mechanical stress, such as the vasculature, bones and joints [17,45,46].

### Loss of peripheral heterochromatin

Similar to chronologically aged normal human cell, progeric cells exhibit loss of transcriptionally repressed and compacted peripheral heterochromatin, as shown by a reduction in histone H3 trimethylation at lysines 9 and 27 (H3K9me3 and H3K27me3) and electron microscopy [23,36,47,48]. Loss of heterochromatin is concomitant with the down-regulation of many proteins involved in epigenetic silencing, including the Enhancer of zeste homolog (EZH2), a member of the polycomb repressive complex (PRC) 2, heterochromatin protein 1 homolog-α (HP1α) and the histone N-methyltransferase Suppressor of variegation 3–9 homolog 1 (SUV39H1) [37,47,49]. Furthermore, a yeast two-hybrid screen using the C-terminal domain of lamin A that is deleted in progerin led to the identification of RBBP4 + 7, both components of the nucleosome remodeling and deacetylase complex (NuRD) and PRC2 complexes that are down-regulated in HGPS cells. Knockdown of both RBBP4 + 7 using siRNA recapitulated some aspects of HGPS, including a reduction in H3K9me3 and elevated levels of DNA damage [49]. Similarly, decompaction of heterochromatin by histone deacetylase inhibitors (HDACi) rendered cells more susceptible to DNA damage and senescence, although it remains unclear whether the observed phenotypes were driven directly by heterochromatin decompaction or other changes caused by HDACi treatment [50,51]. Nevertheless, the precise mechanism how progerin triggers this drastic change in chromatin compaction remains unclear. For instance, a recent transcriptional analysis of control versus progerin-expressing mouse skin found no significant changes in the expression of key epigenetic modulators [52], and expression of progerin in telomerase-positive cells did not result in any transcriptional changes [23].

What is clear is that progerin-induced heterochromatin loss is not a consequence of cells undergoing senescence; as an expression of progerin in immortalized, or telomerase-positive cells, did not prevent heterochromatin decompaction [23,36]. Furthermore, by expressing progerin during different cell cycle stages, it was recently shown that progerin triggers heterochromatin decompaction in growth-arrested (G0) cells, whilst DNA damage accumulates exclusively during DNA replication, thus separating these two phenotypes [48]. By visualizing heterochromatin levels (H3K9me3 and H3K27me3), DNA damage and progerin levels at single-cell resolution, it was also shown that cells with low levels of heterochromatin are more prone to accumulate progerin-induced DNA damage, whilst progerin removal (in G0 cells) restored heterochromatin levels and prevented DNA damage accumulation during subsequent rounds of DNA replication [48,53]. Collectively, these results suggest that chromatin structure plays an important role in causing progerin-induced DNA damage. Interestingly, cells derived from MAD or RD patients exhibit similar chromatin alterations, suggesting that this may be a conserved feature of laminopathies caused by an accumulation of incompletely processed lamin A [54,55]. Nevertheless, this conclusion is largely based on correlative data and it remains unclear whether overexpression of certain chromatin modifiers would restore, or prevent progerin-induced heterochromatin loss (and DNA damage).

### Progerin-induced DNA damage

Lamins have been implicated in both non-homologous end joining and homology directed repair [38,56,57]. Indeed, cells from HGPS patients or primary human cells expressing progerin, as well as cells derived from *Zmpste24*-deficient mice, accumulate DNA damage foci, as judged by 53BP-1 or γH2A-X staining [38,39]. In addition, mouse embryonic fibroblasts derived from *Lmna- or Zmpste24-*deficient mice exhibit genomic instability including increased aneuploidy, chromosome fusions and breaks. Similarly, cells ectopically expressing progerin show an increased frequency of sister chromatid fusions, chromosomal breaks and anaphase bridges [58].

The root cause of this DNA damage has been attributed to many factors, including a delay in recruitment of the DNA repair factor p53-binding protein (53BP-1) to sites of damage, possibly as a result of impaired shuttling into the nucleus [38,59]. In contrast, we recently exposed G0-arrested human fibroblasts expressing progerin to the DNA damaging agent doxorubicin and studied the temporal dynamics of 53BP-1 and γH2A-X recruitment. Surprisingly, the timing of recruitment of these factors to sites of DNA damage were indistinguishable between control and progerin-positive fibroblasts. These results suggest that progerin expression *per se* may not directly affect the DDR. Nevertheless, it must be noted that these experiments were conducted in growth-arrested cells exposed to progerin for only 4 days. Thus, it is conceivable that HGPS cells that constitutively express progerin may exhibit these and other defects [48].

### The nature of progerin-induced DNA damage

**Figure 1.**
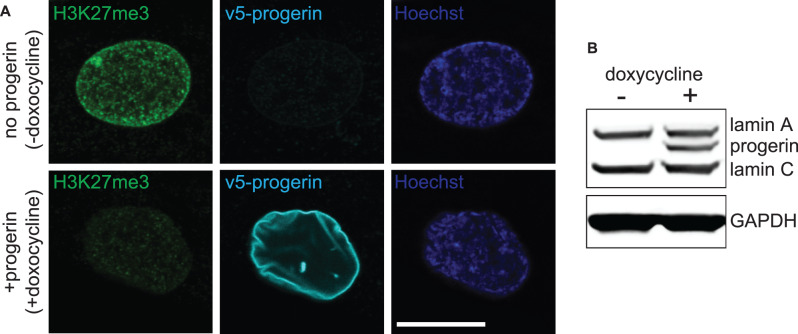
Doxycycline-inducible expression of progerin in proliferating primary human fibroblasts. (**A**) v5-progerin is visualized by anti-v5-antibody (cyan). H3K27me3 staining is indicated in green. Hoechst staining is in blue. Size marker 20 μm. (**B**) Western blot showing doxycyline-dependent expression of progerin. Lamin A, C, progerin and GAPDH are shown.

Increasing evidence suggests that the DNA damage and chronic checkpoint activation in HGPS cells are caused by progerin-induced replication defects. Lamin A/C is required to restart replication forks after hydroxyurea-induced replication stress [60] and progerin-induced DNA damage co-localizes with phospho-RPA32 (ser33) and XPA, proteins that bind to single-stranded DNA at replication forks [61]. Moreover, progerin-induced DNA damage preferentially accumulated in BrdU+ cells, suggesting that damage occurred during S-phase. In contrast, preventing the growth of progerin-expressing cells by serum starvation, or expressing progerin in contact-inhibited (G0) cells (using the doxycycline-inducible system depicted in Figure 1), reduced/prevented the accumulation of DNA damage, respectively. Collectively, these studies link progerin-induced DNA damage to replication fork collapse during S-phase [48,61–63].

But how does progerin affect DNA replication? Previous studies showed that progerin or pre-lamin A expression disrupt the interaction between lamin A and the proliferating cell nuclear antigen (PCNA), a component of replisome that is essential for replication fork progression. This results in sequestration of PCNA away from the replicating fork and exposes the forks’ ds-ssDNA junction to binding by XPA [63,64], whilst reducing progerin levels restored PCNA binding to the fork [61–63]. Single-molecule replication studies using DNA fiber assays provided further evidence that progerin expression triggers replication fork stalling and deprotection. Treatment with Mirin, an inhibitor of the MRE11 nuclease, prevented fork degradation and rescued progerin-induced replication defects [65]. Lastly, by using the aforementioned inducible expression system, we determined the temporal dynamics of DNA damage accumulation during S-phase and found that progerin causes damage exclusively during the late stages of DNA replication prior to chromosome condensation and mitosis [48]. This timing coincides with the replication of telomeres that are located near the nuclear periphery, as well as with the replication of heterochromatin [66,67].

These results imply that progerin-induced DNA damage may only occur in proliferating cells, and raises the question how relevant these cell culture-based findings may be *in vivo*? Although it has been thought that some adult tissues hardly turn over, there is increasing evidence that suggests otherwise [68]. Several studies demonstrated that ∼40% of cardiomyocytes are replaced during a normal lifetime [69,70]. This turnover occurs independent of ploidy and is highest in younger individuals [71]. Additionally, non-cardiomyocyte populations, such as vascular smooth muscle cells from the same hearts exhibited turnover rates of ∼15% per year [70]. Nevertheless, it is likely that DNA replication-independent factors, such as mechanical stress, contribute to progerin-induced DNA damage in some tissues.

### Linking the ends to the periphery

Telomeres are nucleoprotein complexes that cap the physical ends of our linear chromosomes. They consist of 10–20 kb of tandem TTAGGG repeats and are bound by the shelterin complex. Shelterin consists of six proteins that protect the chromosome ends from nucleolytic degradation and illegitimate activation of DNA damage checkpoints. In embryonic, and some adult stem cells, germ cells and ∼85% of cancers, shelterin regulates telomere length homeostasis by recruiting telomerase, a ribonucleoprotein that can elongate telomeres, to the telomere terminus [72]. In somatic cells, telomeres shorten during each replication cycle as a result of the end replication problem. Critically shortened telomeres, or loss of shelterin components elicit a persistent DNA damage checkpoint activation and trigger, depending on cell type, senescence or apoptosis. As such, telomeres represent the Achilles’ heel of the genome and telomere shortening or dysfunction are associated with a wide spectrum of human diseases [73,74].

Telomeres are considered heterochromatic domains and are enriched near the nuclear lamina in both human and mouse cells [23,56,67]. This peripheral localization is elevated during post-mitotic nuclear reassembly [75]. What remains unclear is whether telomeres are actively tethered to the nuclear periphery, or whether they simply co-localize with heterochromatic regions of the genome. In support of a more active tethering model, lamins and associated factors such as the lamina-associated peptide-α (LAP2α) have been shown to co-localize or interact with components of the shelterin complex [23,75–77]. AKTIP, a member of the ubiquitin E2 variant (UEV) enzyme subfamily is enriched at the nuclear periphery and interacts with A- and B-type lamins, shelterin components TRF1 and 2 and PCNA. The depletion of AKTIP in primary human cells recapitulates some aspects of HGPS including replication defects and premature senescence [78].

Cells from HGPS patients have significantly shorter telomeres than their age-matched controls [23,41,42]. As a result, HGPS patient-derived fibroblasts (or cells ectopically expressing progerin) exhibit a limited proliferative capacity and prematurely undergo replicative senescence [23,41,42]. Ectopic expression of hTERT, the catalytic subunit of the telomerase, elongates telomeres in HGPS cells, prevents progerin-induced DNA damage and restores their proliferative capacity [23,58,79,80]. To investigate whether physiological levels of hTERT would be sufficient to prevent progerin-induced defects, progerin was expressed in pluripotent wild type mouse embryonic stem cells (mESC), that express TERT endogenously. Progerin-expressing mESC remained indistinguishable from their wild type counterparts, whilst differentiation of these cells into TERT-negative somatic lineages recapitulated progeric phenotypes [23,81]. Lastly, progerin expression in *TERT*−/− mESC resulted in massive differentiation and cell death [23]. Taken together, both ectopically expressed, or physiological levels of TERT prevent progerin-induced DNA damage and proliferation defects. Nevertheless, the ability of telomerase to prevent progerin-induced DNA damage and proliferation defects may depend on the replicative age of recipient HGPS cells; cells that have undergone senescence, or accumulated excessive genomic instability prior to hTERT transduction may not be rescued. Furthermore, hTERT is not sufficient to prevent these defects in cell that express high levels of progerin (or lamin A), and does not prevent progerin-induced heterochromatin loss or nuclear abnormalities.

What remains unclear is how hTERT prevents, or repairs, progerin-induced DNA damage and what is the physiological relevance of these findings. First, hTERT is active at telomeres only during DNA replication. Thus, the timing of progerin-induced DNA damage (late S-phase) and the ability of hTERT to prevent it coincide. Second, hTERT is expressed during embryonic development and in various adult stem cells, thus rendering these populations more resistant to the detrimental consequences of progerin expression [82]. This contrasts other premature aging syndromes, such as dyskeratosis congenita, that are caused by telomerase dysfunction and result in impaired maintenance of various stem cell compartments [83,84].

### Delineating the chain of events to occur upon progerin expression

**Figure 2.**
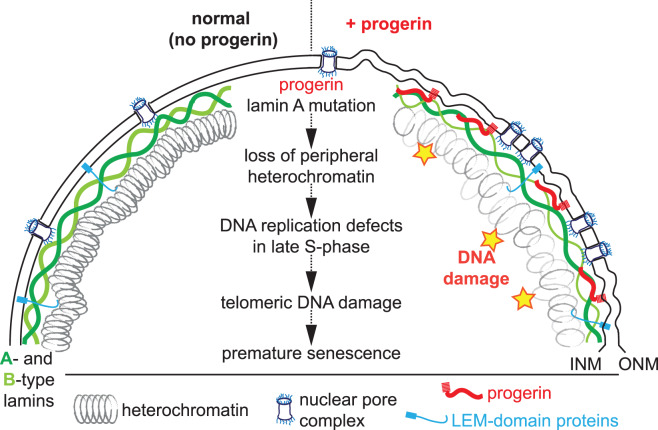
Schematic representation of the nuclear envelope and its associated components in normal (no progerin, left side), or progerin-expressing cells (+ progerin, right side). The nucleus is encapsulated by the inner and outer nuclear membrane (INM, ONM). Cargo is shuttled between the nucleus and cytoplasm by nuclear pore complexes. LEM-domain proteins span the INM and connect it to A- and B-type lamins and peripheral heterochromatin. Progerin retains its farnesyl moiety and remains associated with the INM. Progerin expression results in nuclear abnormalities, heterochromatin decompaction, clustering of nuclear pore complexes, senescence-associated lamin B1 reduction and DNA damage. Center: A speculative model depicting a possible chain of events that commences with an expression of mutant lamin A (progerin), loss of heterochromatin, replication defects and an accumulation of telomeric DNA damage that results in premature cellular senescence.

The usage of isogenic primary and hTERT-immortalized human cell types provides a tool to establish which of the above-mentioned phenotypes are a cause or a consequence of senescence. In addition, inducible expression systems facilitate the restricted expression of progerin to different cell cycle stages and provide information on how different phenotypes may be temporally connected. Based on these experiments, a picture, although incomplete, emerges that delineates the chain of events that occur upon progerin expression and ultimately result in premature senescence (Figure 2). Expression of progerin in contact-inhibited G0-arrested cells results in loss of heterochromatin marks H3K9me3 and H3K27me3, irrespective of hTERT expression, and no accumulation of DNA damage [23,48]. These results are consistent with the reduced number of DNA damage foci in serum-starved HGPS cells [61,63]. The mechanism how progerin results in loss of peripheral heterochromatin remains largely unknown. Upon reinitiating the cell cycle and entering S-phase (by plating the G0-arrested cells sparse), progerin-expressing cells accumulate DNA damage exclusively during the late stages of DNA replications, prior to chromosome condensation and mitosis, and preferentially in cells with low levels of heterochromatin [48,61–63]. Expression of hTERT, or modulation of the DDR specifically at telomeres, prevents (or rescues) progerin-induced DNA damage and senescence [23,48,58,79,85]. In somatic (hTERT-negative) cells, damaged telomeres cannot be repaired by conventional DNA repair processes and, thus, result in a permanent activation of DNA damage checkpoints and premature cellular senescence [23,40,48,58,86]. Removal of progerin in G0-arrested cells leads to a rapid restoration of heterochromatin levels. Moreover, these transiently progerin exposed cells do not exhibit any DNA damage or premature senescence after resuming proliferation. Taken together, these results suggest that progerin removal from quiescent cells or tissues, using morpholino or CRISPR-based approaches is predicted to fully restore their proliferative/regenerative capacity [48,53,87,88].

## Perspectives

- *Highlight the importance of the field*: Premature aging syndromes such as Hutchinson–Gilford progeria provide a unique opportunity to identify and characterize molecular pathways and mechanisms that contribute to human aging.
- *A summary of the current thinking*: Expression of the lamin A mutant progerin results in heterochromatin loss. Cells with low levels of heterochromatin are more prone to develop DNA replication defects and telomeric DNA damage, thereby resulting in premature cellular senescence.
- *A comment on future directions*: Mutations and perturbations of the nuclear lamina give rise to many cellular defects and result in a plethora of human diseases (laminopathies). The key questions are to (1) elucidate the precise mechanism(s) how progerin expression perturbs heterochromatin organization and results in DNA replication defects and damage. (2) Establish how the myriad progerin-induced cellular phenotypes are causally and mechanistically linked. (3) On an organismal level, determine why some mutations exclusively affect certain tissues and how this relates to the disease etiology. Providing answers to these questions will lay the foundation for the development of novel therapeutic interventions.

## Abbreviations

HGPS: Hutchinson–Gilford progeria
INM: inner nuclear membrane
INM: inner nuclear membrane
MAD: mandibuloacral dysplasia
ONM: outer nuclear membrane
PCNA: proliferating cell nuclear antigen
PRC: polycomb repressive complex
